# Melanoma of the Upper Limb and Shoulder: A Surveillance, Epidemiology, and End Results Analysis of Epidemiology and Survival 2000–2019

**DOI:** 10.3390/cancers14225672

**Published:** 2022-11-18

**Authors:** Solange N. Walz, Jérôme Martineau, Matteo Scampa, Daniel F. Kalbermatten, Carlo M. Oranges

**Affiliations:** Department of Plastic, Reconstructive and Aesthetic Surgery, Geneva University Hospitals, Geneva University, 1205 Geneva, Switzerland

**Keywords:** melanoma, upper limb and shoulder, SEER, survival analysis, epidemiology, malignant, dermato-oncology

## Abstract

**Simple Summary:**

Melanoma is a common skin cancer that can occur on any part of the body. The localization of these lesions influences the incidence and overall outcome. The aim of our study was to analyze the epidemiology, treatment patterns, and survival of patients with melanomas arising on the upper extremities and shoulders and to identify potential differences with melanomas on other parts of the body. We report the specific epidemiology of the upper extremities and shoulders and demonstrate a difference from other sites in terms of age, sex, treatment, and survival. This knowledge will help practitioners improve the quality of management of melanoma.

**Abstract:**

(1) Background: Melanoma is the most common life-threatening cancer among skin cancers. Almost all locations of the skin can be affected by melanoma, and the upper limbs are one of the most frequent locations. We aimed to study the epidemiology and survival outcomes of patients with melanoma localized in the upper extremities compared with other sites. (2) Methods: The National Cancer Institute (NCI) Surveillance, Epidemiology, and End Results (SEER) database is considered the most representative of the U.S. population; we extracted melanoma cases diagnosed between 2000 and 2019. Several characteristics, including demographical, pathological, and therapeutic, were recorded, and upper extremity melanomas and melanomas from other areas were compared. Overall survival was assessed, and the groups were compared. (3) Results: 69,436 patients had melanoma in the upper limbs and shoulders and 204,794 in other body parts. Overall, 35,267 patients with upper extremity melanoma were males, 34,169 were females, and the mean age was 60. For the rest of the body, there were 118,654 males and 86,140 females, with a mean age of 59. Surgery alone was the most commonly used treatment, while radiation therapy was the least used for all sites. Women appear to have better survival than men. Superficial spreading melanoma is the least lethal subtype, while nodular melanoma is the most dangerous. (4) Conclusion: Women under 50 are more at risk than men of the same age. The trend reverses after age 50 where men are at greater risk. In addition to gender and age, disease stage and major histologic subtypes influence survival.

## 1. Introduction

Melanoma is responsible for most skin cancer mortality, although it is less common than non-melanoma skin cancers (i.e., basal cell and squamous cell carcinomas). It is estimated that in 2022, 7650 people will die from this cancer in the United States [1]. The exact etiology of melanoma is not entirely understood, but some risk factors have been identified. Genetic factors include low phototype, number of nevi on the body, and positive family history. Environmental factors include high exposure to UV radiation–often suggested by a history of sunburn, particularly in childhood [2,3]. It has been shown that these risks have different outcomes based on the melanoma anatomical sites and the gender of the patient [4,5,6]. For instance, numerous studies have shown that melanomas on the trunk are influenced by sunburn and the number of moles [7,8]. Age is also a risk factor for melanomas of the trunk and the face [8]. However, we did not find studies in which risk factors for upper extremity melanoma were identified [7,8,9].

Nevertheless, these cancers are among the most common in women and men after the lower limbs and trunk, respectively [10]. They also have the worst prognosis, along with the back and scalp [11]. Despite this high prevalence and poor prognosis, there is a paucity of literature on the surveillance and epidemiology of melanoma in hands, arms, and shoulders. The primary objective of this article was to determine the survival and epidemiology of melanoma of the upper limb with the Surveillance, Epidemiology, and End Results (SEER) database, a program of the U.S. National Cancer Institute (NCI) that provides annual data on cancer incidence and survival rates in the U.S. population.

## 2. Materials and Methods

### 2.1. Patient Selection

The NCI Surveillance, Epidemiology, and End Results (SEER) database (https://seer.cancer.gov/, accessed on 10 September 2022) was searched across 17 different registries. Using the third edition of the International Classification of Diseases for Oncology (ICD-O-3/WHO 2008), only cases of “melanoma of the skin” (8720–8790) were selected, and the primary site codes from C44.0 to C44.9 were used to include all cutaneous sites. The database that offers 17 registries (geographical divisions) from 2000 to 2019 was chosen as it covers about 26.5% of the U.S. population [12]. Data were then extracted from the survival section of the SEER*Stat software (version 8.4.0.2). As the SEER program contains public domain data without personal medical identifiers, approval of the Ethics Board was not required.

### 2.2. Variable Selection

The “list of cases” function was selected, and the variables used for our analysis were chosen from this database. These variables included patients’ demographic information (sex, race, marital status, age at diagnosis, and the year of diagnosis) and clinicopathological characteristics (staging, grade, histological subtypes, tumor size, Breslow thickness, mitotic rate, ulceration, surgery of primary site, radiotherapy, survival in months, and vital status). The “merging” tool offered by SEER*stat was used to group the SEER Summary Stages of 1998–2017 and 2004+ to include the disease extension of all selected patients (in situ, localized, regional, and distant). In situ tumors stay in the top layer of the epidermis. According to the Breslow thickness staging, a localized tumor is considered a T1 stage, meaning it stays in the epidermis. Since only 13 patients were classified as in situ and the treatment does not differ significantly between these two stages, we decided to combine the in situ stage with the localized stage for our analysis. The regional stage involves the invasion of the entire dermis and regional lymph node invasion. The distant stage is defined by underlying tissue invasion (bone, cartilage, muscle) and generalized metastatic skin lesions.

The different treatments proposed for melanoma were also merged. We differentiated the type of surgery–less than 1 cm margin, 1–2 cm margins, Mohs surgery, wide resection, and no surgery–and combined it with radiation therapy or no radiation therapy. The survival duration was defined in months from the date of diagnosis to the study cutoff date, preset to December 2019. For a more straightforward interpretation, we also separated patients by age at diagnosis into four equal percentile groups: ≤50 years, 51–61 years, 62–71 years and ≥72 years. Since Breslow thickness records were unavailable until after 2010, we separated the results into a new subgroup to exclude patients registered before 2010. 

### 2.3. Statistical Analysis

Statistical analysis was performed using IBM SPSS version 28 (I.B.M., Armonk, NY, USA) once the data were extracted from the SEER*stat software. In order to compare the upper limb and shoulder data to other cutaneous sites, the chi-square test was employed to identify significant differences across groups. A Z-test was made to compare the data between age groups.

The Kaplan-Meier method was used to determine patient survival and the log-rank test was used to detect differences in survival between subgroups.

A multivariable Cox regression model was used to estimate prognostic factors between sex, age at diagnosis, histological subtypes, stage, treatment sequence, Breslow thickness, ulceration, and mitotic rate. For better understanding, we included only the five most frequent histological subtypes.

We considered a *p*-value less than 0.005 significant since we were analyzing a large number of patients.

## 3. Results

**Figure 1:**
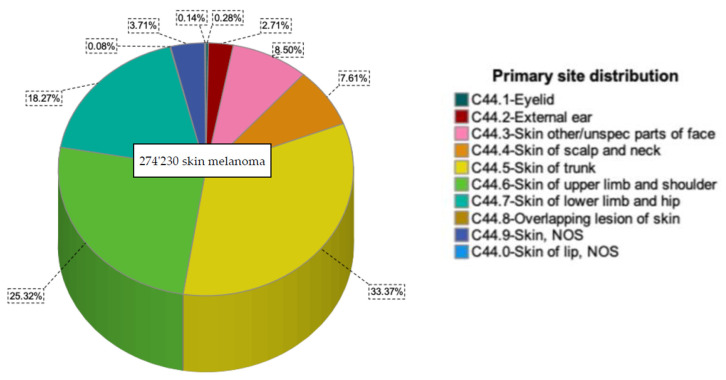
Melanoma distribution according to the primary site.

A total of 69,436 cases of melanoma of the upper limbs and shoulders and 204,794 melanomas of other parts of the body were identified between 2000 and 2019 (Figure 1). Most cases of upper limb melanomas (*n* = 20,627, 29.7%) were diagnosed between 2015 and 2019. Overall, 204,794 cases of melanoma on other parts of the body were recorded between 2000 and 2019. Analogously, most of these cases (*n* = 58,822, 28.7%) were diagnosed between 2015–2019. The general demographic and clinicopathological characteristics of our cohort are listed in Table 1. 

**Figure 2:**
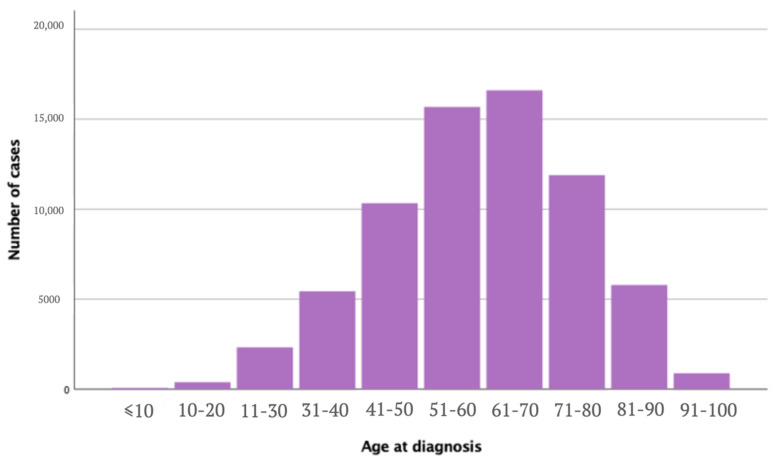
Number of melanomas of the upper limbs and shoulders cases by age group.

The upper limb and shoulder group and the other locations group were significantly different in terms of age, sex, ethnicity, subtypes, stage at diagnosis, and treatment procedures. Age at diagnosis for upper limbs and shoulders melanoma ranged between 0 and 99 years, with a mean age of 60 years old (σ = 15.7), compared to a mean age of 58.9 years old (σ = 16.9) for melanoma on other sites (*p* < 0.005) (Figure 2). Females were more often affected in the upper limbs and shoulders (49.2%) compared to other locations (42.1%) (*p* < 0.005). In comparison, males were more affected in other locations (57.9%) compared to the upper limbs and shoulders (50.8%) (*p* < 0.005). The most affected population were white patients, with the upper limbs and shoulders cohort including 65,287 white patients (94.0%) and 192,416 white patients (94.0%) on other sites. There were 167 cases in black patients (0.24%) with melanoma of the upper limbs and shoulders and 1024 (0.5%) with melanoma in other sites. Overall, 164 patients (0.2%) in the upper limbs and shoulders group were American Indian / Alaska Native patients compared to 522 patients (0.3%) in the other sites group, and 333 patients (0.5%) were Asian or Pacific Islanders in the upper limbs and shoulders cohort and 1513 (0.7%) in the other sites group. Race was unknown for 3485 patients (5,0%) in the upper limbs and shoulders melanoma group and 9319 patients (4.6%) in the other sites.

Eighteen histologic subtypes were described, and the five most frequent subtypes were selected for our survival analysis. The most frequent histology was malignant melanomas, NOS (8720/3), reported in 33,643 patients (48.5%) in the upper limbs and shoulders melanoma group and 103,084 (50.3%) in the other sites melanoma group. The second most frequent histologic subtype was superficial spreading melanoma (*n* = 23,479, 33.81% upper limbs and shoulders, and *n* = 64,158, 31.3% other sites). Nodular melanoma was the third most frequent subtype in the upper limbs and shoulders melanoma group (*n* = 5361, 6.7%) and the other sites group (*n* = 13,289, 6.5%). Patients with upper limbs and shoulders melanoma presented the following stages at diagnosis: localized in 88.02% (*n* = 61,135), regional in 6.8% (*n* = 4725), distant in 1.1% (*n* = 767), and not specified in 4.1% (*n* = 2809). The stage presented at diagnosis in patients with melanoma of other body parts was as follows; localized in 81.1% (*n* = 166,042), regional in 9.5% (*n* = 19,343), distant in 4.8% (*n* = 9900), and not specified in 4.6% (*n* = 9509). 

For upper limbs and shoulders melanoma cases, surgery alone was performed in 64,617 patients (93.1%), and radiation therapy alone was performed in 399 cases (0.6%). Surgery and radiation therapy were combined in 739 patients (1.1%). No surgery or radiation was performed in 3425 patients (4.9%). Of the patients who did not undergo surgery, 2351 (3.4%) did not receive surgery as it was not recommended, 513 did not have surgery when it was recommended for various reasons (refusal, death, unknown). In most cases (86.2%), no regional lymph nodes were removed.

In melanoma on other localization cases, surgery alone was performed in 184,329 patients (90%), and radiation therapy alone was performed in 1183 cases (0.6%). The combination of surgery and radiation therapy was performed in 2403 patients (1.2%). No surgery or radiation was performed in 16,265 patients (7.9%). Of the patients who did not benefit from surgical treatment, 15,069 patients (7.4%) did not undergo surgery as it was not recommended, and 2127 patients (1.3%) did not have surgery when it was recommended for various reasons (refusal, death, unknown).

**Figure 3:**
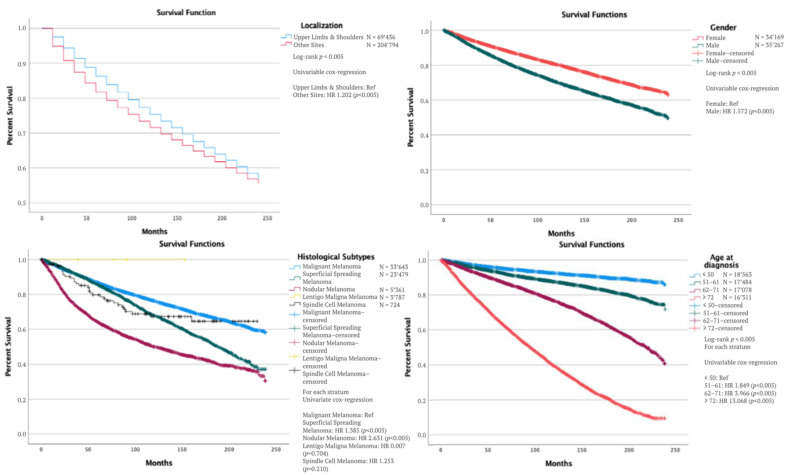
Overall survival according to the primary site, gender, histological subtypes, and age at diagnosis for upper limbs and shoulders melanoma.

Survival analysis (Figure 3) showed that the median overall 5-year survival rate in patients with melanoma on the upper limbs and shoulders was not reached. Overall survival (OS) was 86.5% at five years, 75.5% at 10 years, and 66.1% at 15 years. We found a significant statistical difference when assessing the OS between the four age categories (*p* < 0.005). There was a statistically significantly better OS in females compared to males (*p* < 0.005). 

According to the histological subtype, superficial spreading (ICD-O3 8743/3) was found to have the best OS among all subtypes (*p* < 0.001); followed by lentigo maligna melanoma (ICD-OS8742/3), and spindle cell melanoma (ICD-O3 8772/3). Nodular melanoma (ICD-O3 8721/3) was associated with the worst OS (*p* < 0.005) (Table 2).

Survival was significantly different when comparing disease stages at diagnosis. Patients with localized disease had a significantly better OS than patients with regional disease (*p* < 0.005). The same was observed when comparing the OS in patients with localized and distant diseases (*p* < 0.005).

Most patients were treated with surgery without radiation, and this treatment was associated with the best OS (*p* < 0.005), followed by no surgery and radiation therapy (*p* < 0.005), surgery with radiation, and finally, radiation therapy alone (*p* < 0.005). All subdivisions of Breslow thickness were also significantly different in terms of the OS–the greater the Breslow thickness, the worse the survival. The presence or absence of ulceration impacted patient survival, with a significantly worse OS (*p* < 0.005) associated with the presence of ulceration. The same was observed with the mitosis rate–i.e., the higher the mitosis rate, the worse the OS (Table 3).

## 4. Discussion

Our study identified more than 69,000 melanomas on the upper limbs and shoulders, which is, to our knowledge, the most extensive survival study in this population to date. As observed in other studies, there was a direct linear increase in incidence over the decades: 19.6% of diagnoses were made from 2000 to 2004, 23.9% from 2005 to 2009, 26.9% from 2010 to 2014, and 29.1% from 2015–2020 [13]. Nonetheless, mortality has remained similar over the years. This trend is probably due to increased UV exposure in the general population and a better understanding of the disease that allows earlier diagnosis and better medical management [13,14,15,16].

We found that the incidence of melanoma of upper limbs and shoulders was higher in white men but differed by gender depending on age–with women under 50 being at higher risk than men under 50, and a reversal of this pattern after the age of 50. These results affirm current knowledge [16,17,18,19,20]. This could be related to the emergence of a modern aesthetic standard where tanned skin is considered desirable [21,22]. We found that melanoma on the upper limbs and shoulders was mainly diagnosed at a localized stage, especially in the population under 50 [13,20,23]. Indeed, given that it is a part of the body that can be easily checked, people may notice a change early on and consult when the cancer is still in a primitive stage. Elderly patients were more likely to be diagnosed at regional or distant disease stages and had the worst survival across the four age categories [23].

Although the superficial spreading melanoma type constitutes the majority of melanomas, as in other articles [24,25,26], nodular melanoma was more often diagnosed in the upper arms and shoulders when we separated age groups. In contrast, superficial spreading melanoma was more frequent in melanomas of other sites in people older than 50, but nodular melanoma was still more prevalent in younger people. Nodular melanoma, unlike superficial melanoma, has no particular characteristic, so a visual examination is sometimes not sufficient to detect the disease [13,14,15,26]. This result should be analyzed with caution since the histology of most melanomas has not been identified. Consistent with other studies, superficial spreading melanoma has a better prognosis than nodular melanoma [26,27]. Histologic subtype distinction is conducted according to the 2008 WHO classification. Analyzing subtypes according to their tumoral pathway as described in the WHO 2018 classification was impossible because of the lack of available data. Furthermore, analysis of specific genomic alterations was not performed as they were not reported in the SEER database.

The 5-year overall survival (86.5%) for the upper limbs and shoulders in our analysis is consistent with the American Cancer Society report, which estimates 5-year survival for all sites at around 90% [1]. As mentioned above, the arms are easily accessible for self-monitoring, which may also explain the better survival compared to melanoma at other sites, including scalp melanoma which frequently ulcerates. Unlike melanomas of the scalp, which are at greater risk of metastasizing in the brain, melanomas of the upper extremity are located further away from vital organs [28,29]. Survival in younger people is also significantly higher than in older people. While melanoma survival is reduced in the elderly, it is not necessarily responsible for the death of these patients. In our research on the SEER registry, only 6.3% of deaths were due to complications of melanoma. Even though it is occasionally lethal, melanoma remains one of the co-morbidities among many others accumulated by the elderly. We did not analyze specific survival because we believe it is less representative of real life. Real-world patients, especially elderly patients often have multiple comorbidities that will influence their overall survival. Therefore, we found that studying the influence of melanoma on overall survival rather than disease-specific survival–which is not representative of reality–was more appropriate. 

The latest version of the American Joint Committee on Cancer Classification, which defines the TNM classification of melanoma at initial diagnosis, is a valuable tool to assist the clinician in treatment selection. There are four different stages with specific subgroups, with the gradation of the stage being directly related to the prognosis. The SEER stage used in this study remains comparable to the AJCC classification, with localized disease corresponding to AJCC stages I and II, the regional stage corresponding to stage III, and the distant disease to stage IV. Our results confirm the prognosis is related to the AJCC classification, which notifies decreased survival in the more advanced stages of the neoplasm.

No further investigation is necessary for melanomas smaller than 0.8 mm. As early as stage IB, lymph node sonography is recommended. After stage IIC, or when the tumor is more than 4.0 mm thick with ulceration, a full-body CT or PET-CT scan and a brain MRI are recommended. Screening for mutations is required in stage III, where lymph node invasion is involved. Treatment of melanoma often requires a multidisciplinary team, including dermatologists, oncologists, pathologists, radiologists, and plastic surgeons. 

Depending on the tumor thickness, surgical excision should have a margin of 1 to 2 cm from the tumor [30]. The National Comprehensive Cancer Network recommends an excision between 0.5 and 1 cm for in situ melanoma. A tumor less than 1.0 mm thick requires a 1 cm excision. For tumors thicker than 1.0 to 2.0 mm, an excision of 1 to 2 cm is required. Above 2.0 mm, the surgeon is advised to take 2 cm margins. By following these guidelines, the specific survival of melanoma is significantly improved [31]. 

In most cases, the surgical wound following the excision of a melanoma on the arm or shoulder can be closed directly. However, in some situations–especially when the lesion is too extensive, ulcerated, or deep–performing a skin graft or a flap to prevent damage to the underlying neurovascular structures is necessary [32,33]. These reconstructive techniques are paramount in preventing functional deficits and improving the overall appearance of the surgical wound [34].

Moreover, lymph node dissection is indicated in cases with ulceration where the melanoma is thicker than 1.0 mm or 0.8 mm. When in-transit metastases are present, broader excision and adjuvant therapies may be necessary. Immunotherapy, chemotherapy, and radiotherapy are used for advanced melanoma [35,36].

In our study, more than 93% of our population benefited from surgery as the only form of treatment for melanoma of the upper extremities and shoulders. These results reinforce the current literature where surgery alone is the most common therapeutic procedure employed [15,35,36]. This technique also demonstrated better survival, followed by no surgery and no radiotherapy, surgery with radiotherapy, and radiotherapy alone. These results should be interpreted with caution. Indeed, an absence of treatment does not inevitably indicate better survival; instead, these people have been recently diagnosed and not yet treated. In a similar perspective, although radiotherapy can be used at very early stages when surgical treatment is not an option, it is primarily employed in the late stages of the disease and for recurrence cases linked to lower survival [37,38]. Chemotherapy, targeted therapy, and immunotherapy are other treatments used for patients with advanced melanoma. Unfortunately, the SEER database does not provide information on those treatments, and therefore the analysis could not be performed [39]. Furthermore, the analysis of treatment efficiency is limited by the absence of data regarding tumoral relapse. AJCC stage I and II was found to be associated with loco-regional relapse, whereas stage III provoked distant relapse [40]. Histopathological subtypes such as nodular melanoma or acral lentiginous melanoma were associated with more aggressive histopathological characteristics such as deeper Breslow, presence of ulceration, or lymphovascular invasion, resulting in a higher relapse rate [41,42].

The lack of Breslow report before 2010 represents a significant limitation of this study, limiting multivariable and Breslow analysis to half the study population.

## 5. Conclusions

Melanoma on the upper limbs and shoulders are mainly diagnosed at a localized stage and primarily affect women under 50 and men over 50. The expected survival is principally influenced by age, the pathological stage at diagnosis, and the main histological subtypes. Surgery is still the primary treatment for early disease management and has the highest overall survival. 

Treatment modalities for advanced stages should be evaluated for survival and disease recurrence, including chemotherapy, targeted therapy, and immunotherapy.

## Figures and Tables

**Table 1:** Demographic, pathological, and treatment characteristics of the study population.

	Upper Limb and Shoulder *n* (%)	Other Sites *n* (%)	*p*-Value	Total *n* (%)
Total	69,436 (100)	204,794 (100)		274,230 (100)
Age at diagnosis [years]			<0.005	
Mean (SD)	60.00 (15.7)	58.86 (16.9)		
Median	61	60		
Age group			<0.005	
≤50	18,563 (26.7)	63,946 (31.2)		82,509 (30.1)
51–61	17,484 (25.2)	49,863 (24.3)		67,347 (24.6)
62–71	16,311 (23.5)	43,435 (21.2)		59,746 (21.8)
≥72	17,078 (24.6)	47,550 (23.2)		64,628 (23.6)
Gender			<0.005	
Male	35,267 (50.8)	118,654 (57.9)		153,921 (56.1)
Female	34,169 (49.2)	86,140 (42.1)		120,309 (43.9)
Ethnicity			<0.005	
White	65,287 (94)	192,416 (94)		257,703 (94)
Black	167 (0.2)	1024 (0.5)		1191 (0.4)
Asian or Pacific Islander	333 (0.5)	1513 (0.7)		1846 (0.7)
American Indian/Alaska Native	164 (0.2)	522 (0.3)		686 (0.3)
Unknown	3485 (5.0)	9319 (4.6)		12,804 (4.7)
Year of Diagnosis (%)			<0.005	
2000–2004	13,613 (19.6)	42,737 (20.9)		(20.6)
2005–2009	16,546 (23.8)	49,278 (24.2)		65,824 (24)
2010–2014	18,650 (26.9)	53,957 (26.4)		72,607 (26.5)
2015–2019	20,627 (29.7)	58,822 (28.7)		79,449 (29)
Histologic Subtypes (%)			<0.005	
8720/3: Malignant Melanoma, NOS	33,643 (48.5)	103,084 (50.3)		136,727 (49.9)
8743/3: Superficial Spreading Melanoma	23,479 (33.8)	64,158 (31.3)		87,637 (32)
8721/3: Nodular Melanoma	5361 (7.7)	1363 (6.7)		18,999 (6.9)
8742/3: Lentigo Maligna Melanoma	3787 (5.5)	13,289 (6.5)		17,076 (6.2)
8772/3: Spindle cell melanoma, NOS	724 (1)	2076 (1)		2800 (1)
8745/3: Desmoplastic melanoma, malignant	689 (1)	2173 (1.1)		2862 (1)
8744/3: Acral lentiginous melanoma, malignant	471 (0.7)	2270 (1.1)		2741 (1)
8730/3: Amelanotic melanoma	303 (0.4)	707 (0.4)		1010 (0.4)
8723/3: Malignant melanoma, regressing	299 (0.4)	890 (0.4)		1189 (0.4)
8761/3: Malignant melanoma in giant pigmented nevus	204 (0.3)	727 (0.4)		931 (0.3)
8740/3: Malignant melanoma in junctional nevus	194 (0.3)	677 (0.3)		871 (0.3)
8771/3: Epithelioid cell melanoma	135 (0.2)	506 (0.2)		641 (0.2)
8770/3: Mixed epithelioid and spindle cell melanoma	113 (0.2)	470 (0.2)		583 (0.2)
8722/3: Balloon Cell Melanoma	18 (0.03)	67 (0.03)		85 (0.03)
8780/3: Blue nevus, malignant	10 (0.01)	27 (0.01)		37 (0.01)
8741/3: Malignant melanoma in precancerous melanosis	4 (0.01)	12 (0.01)		16 (0.01)
8727/3: Dysplastic nevus, malignant	1 (0.00)	0 (0.00)		1 (0.0)
8773/3: Spindle cell melanoma, type A	1 (0.00)	7 (0.00)		1 (0.0)
Stage (%)			<0.005	
Localized	61,135 (88.02)	166,042 (81.1)		227,177 (82.8)
Regional	4725 (6.8)	19,343 (9.5)		24,068 (8.8)
Distant	767 (1.1)	9900 (4.8)		10,667 (3.9)
Other/not specified	2809 (4.1)	9509 (4.6)		12,318 (4.5)
Type of Surgery (%)			<0.005	
No Surgery or Radiation	3425 (4.9)	16,265 (7.9)		19,690 (7.2)
Radiation Only	399 (0.6)	1183 (0.6)		1582 (0.6)
Surgery with Radiation	739 (1.1)	2403 (1.2)		3142 (1.1)
Surgery without Radiation	64,617 (93.1)	184,329 (90.0)		248,946 (90.8)
Other/Not specified	256 (0.4)	614 (0.3)		870 (0.3)
Reason for no surgery (%)			<0.005	
Not recommended	2341 (3.4)	14,886 (7.3)		17,227 (6.3)
Recommended but not performed; patient refused	39 (0.1)	217 (0.1)		217 (0.1)
Recommended but not performed, unknown reason	474 (0.7)	1879 (0.9)		1879 (0.9)
Not performed; patient died prior to recommended surgery	7 (0.01)	31 (0.02)		38 (0.01)
Recommended, unknown if performed	185 (0.3)	575 (0.3)		760 (0.3)
Not recommended, contraindicated due to other cond; autopsy only (1973–2002)	10 (0.01)	183 (0.1)		193 (0.1)
Surgery performed	66,208 (95.4)	186,428 (91.0)		252,636 (92.1)
Unknown; death certificate; or autopsy only (2003+)	172 (0.3)	595 (0.3)		767 (0.3)
Lymph Node Removal			<0.005	
No Regional Lymph Nodes Removed	59,877 (86.2)	174,517 (85.2)		234,394 (85.5)
Regional Lymph Nodes Removed	4244 (6.1)	14,939 (5.4)		19,183 (7.0)
Sentinel Nodes Removed	4653 (6.7)	12,692 (4.6)		17,345 (6.3)
Unknown	662 (1.0)	2646 (1.0)		3308 (1.2)
Ulceration ^1^			<0.005	
Ulceration not identified	32,548 (46.87)	88,395 (43.16)		120,943 (44.10)
Ulceration present	4686 (6.71)	13,853 (6.76)		18,509 (6.75)
Not documented	32,323 (46.42)	102,546 (50.07)		134,778 (49.15)
Breslow Thickness (mm) ^1^			<0.005	
<0.8 mm	21,773 (59.0)	58,615 (58.5)		80,388 (58.6)
0.8 to 1 mm	4212 (11,4)	11,603 (11.0)		15,275 (11.1)
>1 to 2 mm	5398 (14.6)	14,265 (14.2)		19,663 (14.3)
>2 to 4 mm	3113 (8.4)	8845 (8.8)		11,958 (8.7)
>4 mm	2384 (6.5)	7465 (7.4)		9849 (7.2)
No mass found	29 (0.1)	2601 (2.5)		2630 (1.9)

^1^ results only from 2010 to 2019.

**Table 2:** Comparison between age groups.

	Upper Limbs & Shoulders				Other Sites			
	≤50	51–61	62–71	≥72	≤50	51–61	62–71	≥72
Gender (%)								
Female	11,280 (33.0)	8372 (24.5)	6954 (20.4)	7563 (22.1)	35,211 (40.9)	19,449 (22.6)	14,537 (16.9)	16,943 (19.7)
Male	7283 (20.7)	9112 (25.8)	9357 (26.5)	9515 (27.9)	28,735 (24.2)	30,414 (25.6)	28,898 (24.4)	30,607 (25.8)
Histological Subtypes (%)								
Malignant Melanoma	9252 (27.5)	8529 (25.4)	7810 (23.2)	8052 (23.9)	32,145 (31.2)	23,393 (24.6)	21,872 (21.2)	23,674 (23.0)
Superficial Spreading Melanoma	266 (7.0)	765 (20.2)	1205 (31.8)	1551 (41.0)	1208 (9.1)	2544 (19.1)	2963 (21.7)	4285 (31.4)
Nodular Melanoma	1029 (19.2)	1184 (22.1)	1181 (22.0)	1967 (36.7)	3239 (23.7)	3151 (23.1)	2963 (21.7)	4285 (31.4)
Lentigo Melanoma	0 (0.0)	1 (25.0)	1 (25.0)	2 (50.0)	5 (41.7)	2 (16.7)	2 (16.7)	3 (25.0)
Spindle Cell Melanoma	43 (38.1)	15 (13.3)	27 (23.9)	28 (24.8)	183 (38.9)	96 (20.4)	78 (16.6)	113 (24.0)
Cause of death (%)								
Dead attributable to causes other than melanoma	437 (4.4)	1001 (10.0)	2095 (20.9)	6468 (64.7)	1400 (5.2)	2908 (10.8)	5450 (20.2)	17,260 (63.9)
Dead attributable to melanoma	843 (19.2)	880 (20.0)	1010 (23.0)	1665 (37.9)	4761 (20.7)	5240 (22.8)	5116 (22.3)	7863 (34.2)
Other (alive/unknown)	17,283 (31.4)	15,603 (28.4)	13,206 (24.0)	8945 (16.3)	57,785 (37.3)	41,715 (26.9)	32,869 (21.2)	22,427 (14.5)

**Table 3:** Multivariate Analysis.

				95%CI for Exp(B)	
	B	Sig	Exp (B)	Lower	Upper
Age at diagnosis ^a^					
≤50					
51–61	0.611	<0.005	1.822	1.788	1.898
62–71	1.197	<0.005	3.310	3.217	3.405
≥72	2.265	<0.005	9.629	9.386	9.879
Gender ^b^					
Female					
Male	0.322	<0.005	1.380	1.357	1.402
Main histological Subtypes ^c^					
8720/3: Malignant Melanoma					
8743/3: Superficial Spreading Melanoma	−0.320	<0.005	0.726	0.704	0.749
8721/3: Nodular Melanoma	0.648	<0.005	1.912	1.867	1.959
8742/3: Lentigo Maligna Melanoma	0.319	0.435	1.375	0.618	3.061
8772/3: Spindle Cell Melanoma	0.379	<0.005	1.460	1.272	1.677
Other	−0.290	<0.005	0.748	0.735	0.762
Stage ^d^					
Localized					
Regional	0.581	<0.005	1.789	1.690	1.893
Distant	1.622	<0.005	5.063	4.655	5.506
Treatment sequence ^e^					
Surgery with radiotherapy					
Surgery without radiotherapy	−0.461	<0.005	0.630	0.591	0.672
No surgery with radiotherapy	0.500	<0.005	1.649	1.531	1.776
No surgery without radiotherapy	−0.125	<0.005	0.883	0.827	0.942
Breslow ^f^					
<0.8 mm					
0.8 to 1 mm	0.224	<0.005	1.251	1.162	1.348
>1 to 2 mm	0.656	<0.005	1.927	1.823	2.037
>2 to 4 mm	1.085	<0.005	2.960	2.799	3.131
>4 mm	1.722	<0.005	5.594	5.295	5.911
Ulceration ^f^					
No					
Yes	1.010	<0.005	2.747	2.638	2.859
Mitotic Rate ^f^					
2 or less					
3 to 10	0.885	<0.005	2.422	2.305	2.546
11 or more	1.222	<0.005	3.394	3.170	3.634

^a^ Adjusted for gender, histological subtype. ^b^ Adjusted for age, histological subtype. ^c^ Adjusted for age, gender. ^d^ Adjusted for age, sex, histological subtype, Breslow, ulcer, mitotic rate. ^e^ Adjusted for age, sex, histological subtype, stage. ^f^ Adjusted for age, sex, histological subtype.

## Data Availability

Not applicable.
